# State-of-the-Art Review on the Application of Membrane Bioreactors for Molecular Micro-Contaminant Removal from Aquatic Environment

**DOI:** 10.3390/membranes12040429

**Published:** 2022-04-15

**Authors:** My-Linh Nguyen, Ali Taghvaie Nakhjiri, Mehnaz Kamal, Abdullah Mohamed, Mohammed Algarni, Subbotina Tatyana Yu, Fu-Ming Wang, Chia-Hung Su

**Affiliations:** 1 Graduate Institute of Applied Science and Technology, National Taiwan University of Science and Technology, Taipei City 106335, Taiwan; nguyenmylinh011293@gmail.com (M.-L.N.); mccabe@mail.ntust.edu.tw (F.-M.W.); 2 Department of Petroleum and Chemical Engineering, Science and Research Branch, Islamic Azad University, Tehran 1477893855, Iran; 3 Department of Pharmaceutical Chemistry, College of Pharmacy, Prince Sattam Bin Abdulaziz University, Al-Kharj 11942, Saudi Arabia; mehnaz@gmail.com; 4 Research Centre, Future University in Egypt, New Cairo 11845, Egypt; mohamed.a@fue.edu.eg; 5 Mechanical Engineering Department, Faculty of Engineering, King Abdulaziz University, P.O. Box 344, Rabigh 21911, Saudi Arabia; malgarni1@kau.edu.sa; 6 South Ural State University, 454080 Chelyabinsk, Russia; subbotinati@susu.ru; 7 Department of Chemical Engineering, Ming Chi University of Technology, New Taipei City 243303, Taiwan

**Keywords:** aquatic environment, micro-contaminants, molecular removal, membrane bioreactor

## Abstract

In recent years, the emergence of disparate micro-contaminants in aquatic environments such as water/wastewater sources has eventuated in serious concerns about humans’ health all over the world. Membrane bioreactor (MBR) is considered a noteworthy membrane-based technology, and has been recently of great interest for the removal micro-contaminants. The prominent objective of this review paper is to provide a state-of-the-art review on the potential utilization of MBRs in the field of wastewater treatment and micro-contaminant removal from aquatic/non-aquatic environments. Moreover, the operational advantages of MBRs compared to other traditional technologies in removing disparate sorts of micro-contaminants are discussed to study the ways to increase the sustainability of a clean water supplement. Additionally, common types of micro-contaminants in water/wastewater sources are introduced and their potential detriments on humans’ well-being are presented to inform expert readers about the necessity of micro-contaminant removal. Eventually, operational challenges towards the industrial application of MBRs are presented and the authors discuss feasible future perspectives and suitable solutions to overcome these challenges.

## 1. Introduction

**Figure 1:**
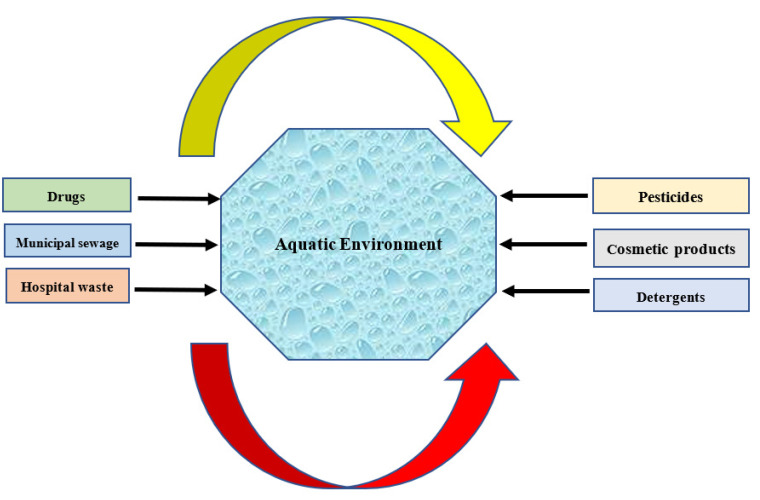
Principal sources of micro-contaminants in aquatic environments.

Global scarcity of water has highlighted the necessity of developing promising wastewater treatment technologies to remove its toxic/detrimental micro-contaminants [1,2]. In recent years, the global accessibility of clean water resources has significantly decreased owing to the existence of some important parameters such as population growth, agricultural-/industrial-based activities and humans’ tendency to live in urban areas. These factors have improved the motivation of scientists all over the world to explore promising water sources [3]. Abnormal distribution of toxic/detrimental micro-contaminants to the aquatic environment without appropriate treatment may eventuate in the occurrence of serious impacts on humans’ health [4,5,6,7,8]. Micro-contaminants (i.e., pesticides, cosmetic devices, detergents, drugs and food additives) are a wide classification of chemical materials existing in the environment at very trace concentrations in the range of ng L^−1^ to µg L^−1^. The European Union (EU) has reported that more than 100,000 chemical substances can be labeled as micro-contaminants and from 30,000 to 70,000 of them are of daily application in disparate activities [9]. Figure 1 schematically demonstrates the principal sources of micro-contaminants in aquatic environments.

**Figure 2:**
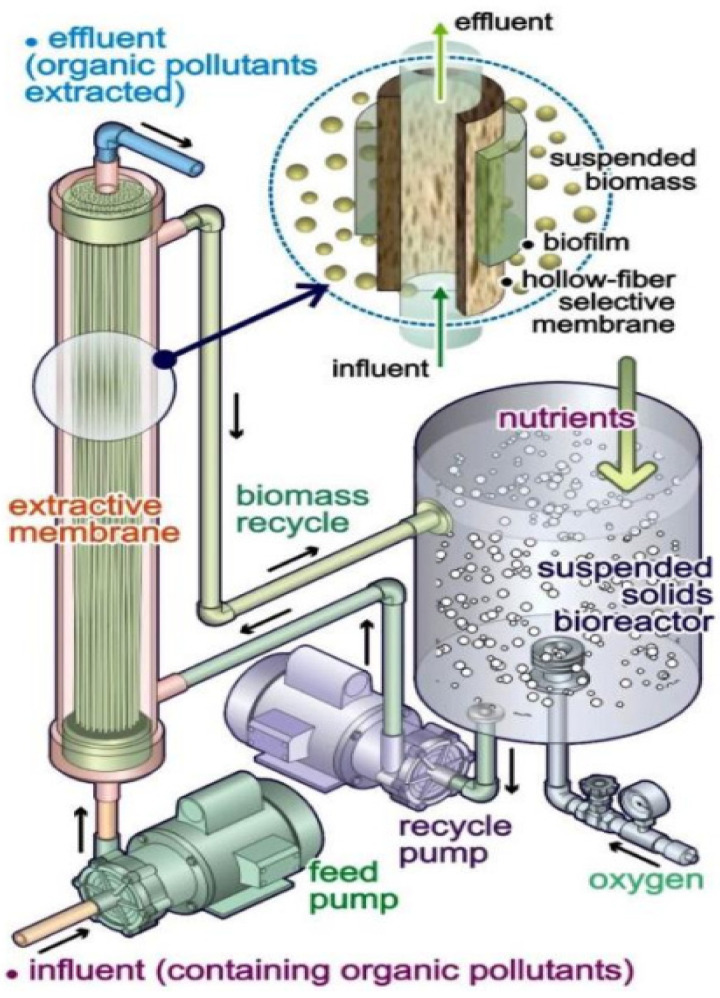
Schematic illustration of micro-contaminant removal using an extractive membrane bioreactor (EMBR). Reprinted from Ref. [25], Copyright (2020), with permission from Elsevier.

Several physicochemical/advanced oxidation (AO) procedures including adsorption on activated carbon, nanofiltration, membrane separation, liquid–liquid extraction, photo-catalytic degradation and photo-oxidation have been recently under evaluation to remove micro-contaminants from aquatic/gaseous environments [10,11,12,13,14,15,16,17,18,19]. Despite the noteworthy privileges of the abovementioned techniques to remove prevalent micro-contaminants from water/waste water sources, they suffer from some undesirable drawbacks such as high energy demand, generation of secondary sludge disposal and the need of poisonous chemical materials [10,20]. Therefore, the development of more promising approaches towards removing micro-contaminants from aquatic environments is of great interest. In the preceding three decades, membrane bioreactors (MBRs) have been well identified as a novel and promising technology to remove different types of micro-contaminants from aquatic environments [21,22]. During these years, MBRs have found great potential of application in disparate industrial operations, especially wastewater treatment and micro-contaminant removal [23,24]. The schematic demonstration of the micro-contaminants’ removal process in an MBR is depicted in Figure 2.

In recent decades, membranes have shown their great potential to remove different types of micro-contaminants from water/wastewater sources. Water treatment processes apply different types of membranes such as microfiltration (MF), ultrafiltration (UF), reverse osmosis (RO) and nanofiltration (NF) membranes. MF membranes possess the greatest pore size and usually separate large particles and microorganisms. UF membranes possess smaller pores in comparison with MF membranes and thus, apart from big particles/microorganisms, have the capability to separate bacteria and soluble macromolecules (i.e., proteins). RO membranes are non-porous. Hence, they have brilliant potential of application to remove various low-molar-mass species like salt ions and organic micro-contaminants. NF membranes are related to a new classification of membranes called “loose” reverse osmosis membranes, which unlike RO membranes, can act at low pressure and suggest selective solute rejection according to both size and charge [26,27].

MBRs can combine biological treatment process and membrane-based separation approach. Sometimes, chemical materials are involved in MBR systems to improve their efficiency [28,29,30,31]. In industrial-associated activities, the operational efficiency of MBRs for micro-contaminant removal has been under accurate investigation since the early 1990s when the installation of the first large-scale MBR was conducted in the United States [32]. Recently, MBRs have achieved great popularity as a reliable alternative for conventional activated sludge (CAS) treatment. MBRs have been able to solve the settleability challenge and unfavorable biomass regeneration by eliminating the clarifier in CAS and its substitution with membrane. MBRs also possess noteworthy characteristics such as high efficiency in removing micro-contaminants from water/wastewater sources, great ability to resist high organic loading and the generation of low amounts of sludge [33,34,35,36,37]. High-quality treated water with a negligible number of micro-contaminants achieved from MBRs may be re-applied for heat integration and process engineering. Very low values of micro-contaminants can hinder the functional failure of sensitive apparatuses or pipes [38,39,40]. Despite the presence of numerous advantages, membrane fouling can be identified as one of the most important operational challenges in MBRs. The occurrence of membrane fouling inside the MBRs results in the contamination of microorganisms and also considerable decrement of the membrane performance [36,41,42,43,44]. Therefore, finding promising ways to decline this problem is of great importance.

The main purpose of this review paper is to discuss the advantages of MBRs compared to other conventional techniques to remove disparate sorts of micro-contaminants in aquatic environments to increase the sustainability of clean water supplement. As a novelty, prevalent types of micro-contaminants in water/wastewater sources are discussed and their potential detriments on humans’ health are presented to inform expert readers about the necessity of micro-contaminant removal. Additionally, operational challenges/constraints towards the industrial application of MBRs are presented and appropriate solutions to overcome these challenges are presented to highlight the future outlook.

## 2. Various Types of Micro-Contaminants in Water/Wastewater Sources and Their Potential Detriments for Health

Pesticides, disinfectants, detergents, triclosan, personal care products, non-steroidal anti-inflammatory (NSAID) drugs (i.e., ibuprofen), sulfamethoxazole and carbamazepine have been recently identified as the micro-contaminants that appear most in ground and surface water/wastewater resources [10,45]. Pollution of ground and surface water/wastewater resources with disparate sources of micro-contaminants is often due to the sewer systems, ground-surface water interaction inside the soil and the polluted water permeation from agricultural lands. It has been reported that the value of micro-contaminants in ground water is lower than this amount in surface water [46]. Table 1 represents comprehensive data about the most prevalent identified micro-contaminants accompanied by their average concentration in wastewater/surface water sources. The concentration amount of the abovementioned micro-contaminants in wastewater sources can fluctuate due to various parameters such as generation rate, application of products, excretion rate and climatic situations [47].

Most micro-contaminants dispersed in the aquatic environment are significantly detrimental and result in serious genotoxicity/mutagenicity in humans/animals because of their non-biodegradable nature [48,49]. For example, successive distribution of endocrine disrupting compounds (EDCs) in surface-/underground water sources eventuates in serious reproduction abnormalities among various types of marine species [50]. Moreover, abnormal increment of antibiotic-resistant microorganisms in the aquatic/non-aquatic environment can be considered as another challenge. Global augmentation in the application of various sorts of antibiotics has caused the appearance of antibiotic-resistant species in disparate environmental matrices [40,43,44]. It is believed that the distribution of toxic micro-contaminants in the aquatic environment will be enhanced in the future because of the increasing rate of population and strong dependency on pharmaceutics. Table 2 enlists the potential detriments of micro-contaminants on the environment.

Scientific investigations about the emergence of macroplastics and microplastics in marine/oceanic environments have been of paramount attention in the recent decades [72,73]. The first evidence about the presence of plastic contamination in marine/oceanic environments was obtained in the 1970s. Based on a report by Carpenter et al., the plastic concentration (mainly cigarette holders) was approximately 3500 items/km^2^ [74]. Recently, numerous studies have been conducted to evaluate the amount of plastic contamination in aquatic environments, particularly rivers and lakes. For instance, Andrady proved that about eighty percent of the plastic contamination in aquatic environments originated from the terrestrial environment [75]. In 2019, Horton et al. perceived that the amount of macroplastic pollution in the terrestrial medium was from 4 to 23 times greater than their existence in marine ecosystems [76]. Freshwater can be identified as the most prominent sources of macroplastics/microplastics flowing into the seas and rivers. Additionally, it can be regarded as a noteworthy transport vector of plastic wastes from terrestrial sources [77]. Therefore, conducting more theoretical/experimental investigations towards studying some important parameters like freshwater ecosystems, main sources of the macroplastics/nanoplastics and dispersion dynamics is of great importance.

## 3. Different Technologies towards Micro-Contaminants Removal

Disparate physicochemical/biological techniques have been recently evaluated to separate micro-contaminants from surface-/underground water sources. Coagulation–flocculation and activated carbon adsorption (ACA) are two procedures that have demonstrated great efficiency in removing micro-contaminants [78,79,80,81]. Biological methods, including MBRs, activated sludge and constructed wetland, are another classification of micro-contaminant removal technologies, which have gained significant popularity in recent years due to their positive advantages such as cost-effectiveness and eco-friendly characteristics [82,83,84,85,86,87]. Hybrid methods, which consider the combination of biological and physicochemical techniques, are the most novel approaches that have been able to open new horizons towards the removal of micro-contaminants from aquatic environments including surface-/underground water sources. This section aims to review the abovementioned procedures to highlight the advantages/disadvantages of each approach in the field of wastewater treatment and micro-contaminant removal technologies [6,54,55].

### 3.1. Prevalent Physico-Chemical Treatment Procedures for Micro-Contaminant Removal

#### 3.1.1. ACA Technique

**Figure 3:**
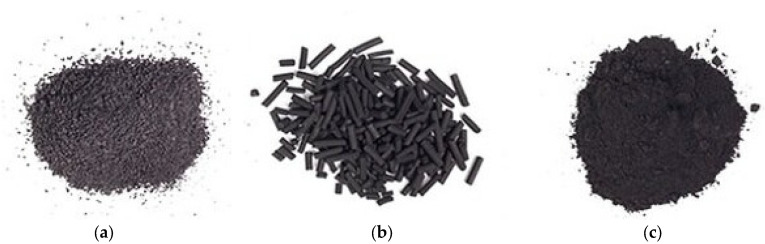
Representation of (**a**) granular, (**b**) pelletized and (**c**) powdered types of activated carbons. Reprinted with permission from Ref. [92].

ACA is one of the most important adsorption-based techniques to remove different types of micro-contaminants from aquatic environments. This process is conducted by the diffusion of contaminants on the surface and after that on the micropores of the activated carbon. Due to the implementation of the diffusion process, hydrophobic contaminants (i.e., toluene and chlorinated solvents) demonstrate superior removal performance compared to the hydrophilic/highly water-soluble contaminants [88,89,90,91]. EDCs, pharmaceutical components (such as antibiotics) and xenobiotic compounds are the most common micro-contaminants that possess the potential of removal using the ACA technique. The removal process of micro-contaminants using the ACA method relies on different parameters, like particle size and pH/concentration of micro-contaminants. Figure 3 illustrates granular, pelletized and powdered types of activated carbons applied to remove micro-contaminants from surface-/underground water sources.

#### 3.1.2. Coagulation–Flocculation (G-F) Technique

This method is identified as a promising adsorption-based technique, which applies colloidal particles (usually named as coagulants) to remove various types of micro-contaminants. Overall, this technique possesses a brilliant capability to remove the existing suspended solids/organic substances in aquatic environments (water and wastewater sources) by adding metal salts/hydroxides (i.e., iron or aluminum salts) [93,94,95,96,97]. The removal process of micro-contaminants from surface-/underground water sources takes place by their adsorption on the surface of metal hydroxide and their consequent collection in the form of sludge for more treatment processing [98]. The removal efficiency of the micro-contaminants on the surface of metal hydroxide depends on their physicochemical properties. Generally, hydrophobic micro-contaminants have better potential to be removed by the G-F technique. To put the issue into perspective, various sorts of micro-pollutants, such as polycyclic aromatic components and humic acid, have satisfactory potential of removal using the G-F technique. Despite the presence of noteworthy privileges, insufficient removal of micro-contaminants with low sorption capability is the principal drawback of the G-F technique [99]. Table 3 represents information about the removal process of micro-contaminants from various wastewater sources applying the G-F technique.

### 3.2. Biological Treatment Procedures

#### 3.2.1. Advanced Oxidation Processes (AOPs)

The existence of great chemical stability and negligible biodegradability of an extensive range of micro-contaminants has reduced their appropriate removal treatment from aquatic environment. Alteration of micro-contaminants to their less poisonous structures occurred using chemical oxidation processes (CAPs). AOPs are known as a well identified class of chemical oxidation processes that have shown their great efficiency in the removal of disparate sorts of micro-contaminants [105]. The categorization of AOPs can take place by the production of hydroxyl and sulfate free radicals [106]. Compared to other CAPs, AOPs are corroborated to be the most efficacious technique in producing hydroxyl radicals, which results in decreasing the amount of organic micro-contaminants in aquatic environments. The non-selectivity of AOPs in the oxidation process of micro-contaminants is one of the privileges of this technique. On the other hand, the emergence of hydroxyl scavenging species in wastewater sources eventuates in decreasing the accessibility of oxidants for micro-contaminant degradation and consequently their removal performance [99,107].

#### 3.2.2. Constructed Wetland (CW) Technique

The CW technique, as a promising environmental-based treatment procedure, has shown great potential of application for removing disparate types of micro-contaminants from aquatic environments in rural areas of third-world countries [108,109]. Recent investigations have corroborated that the CW technique (relying on its structure, configuration and feature), can be effective in removing micro-contaminants from an extensive range of wastewater sources such as domestic/agricultural wastewater and industrial effluent [110,111]. In recent years, the CW method has been of great interest in terms of being applied for removing micro-contaminants from wastewater treatment plants (WWTPs) due to its affordability and excellent adaptability. CW possesses great potential to remove organic/inorganic micro-contaminants such as detrimental pathogens from WWTP effluents, and plays an important role in improving the quality of water and maintaining the ecological environment of aquatic environments [112,113].

#### 3.2.3. Hybrid Reactor System (HRS)

**Figure 4:**
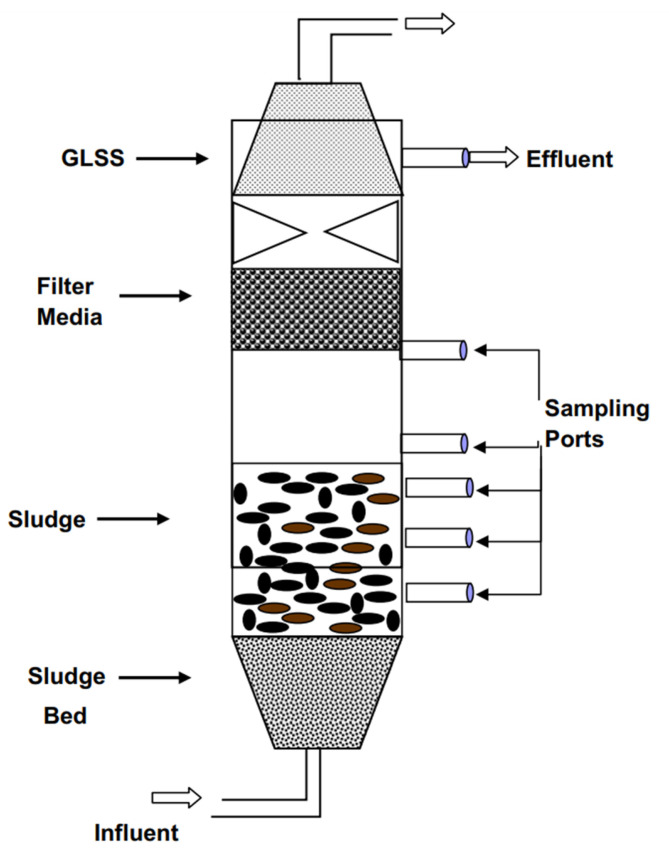
Schematic depiction of an anaerobic HRS. Reprinted from Ref. [116], Copyright (2013), with permission from Elsevier.

HRS is another novel and promising technology to remove micro-pollutants from disparate aquatic/non-aquatic environments. In HRS, a combination of biological and chemical processes takes place to enhance the performance of the removal process. This system possesses great efficiency in removing EDCs and pharmaceutical micro-contaminants by biological processes and chemical-based micro-contaminants such as pesticides and personal care products by chemical processes (i.e., activated carbon based adsorption technique) [55,80,81,114]. Therefore, it is worth noting that the combination of biological and chemical processes can form hybrid treatment systems, which is of great attraction for removing the emergent micro-contaminants in water/wastewater sources. As an example, a hybrid system containing AOP accompanied by a biological treatment procedure has been reported to be significantly efficacious in removing Beta blockers and pesticides with the removal percentage of approximately 100% [99,115]. A schematic demonstration of an anaerobic HRS is presented in Figure 4.

#### 3.2.4. Membrane Bioreactors (MBRs)

Despite the acceptable efficiency of conventional treatment techniques to mitigate the amount of different micro-contaminants (i.e., organic materials and food products) from aquatic environments, they have not been able to demonstrate appropriate performance in the removal of pharmaceutical micro-contaminants. As a result, these detrimental components appear in surface-/ground water sources and significantly endanger humans’ well-being [117,118]. Pharmaceutical components are defined as biologically recalcitrant materials, which means their break down in the environment takes a long time. In doing so, the development of efficient techniques to remove pharmaceutical components (even at trace levels) from aquatic environments is of great importance. In recent years, MBRs have been extensively applied due to their great efficiency in removing various micro-contaminants [119,120]. MBRs have shown their great potential in the removal of numerous organic/inorganic micro-contaminants due to having three privileges such as excellent adsorption capacity, great sludge biodegradation and suitable efficiency in removing different types of micro-contaminants adsorbed on rejected particles inside the membrane [121,122]. It has been reported that the removal efficiency of micro-contaminants applying MBR systems is much higher than the other technologies due to the existence of a microbial population in the proximity of the membrane surface [99]. Table 4 presents the removal efficiency of different sorts of pharmaceutical micropollutants using MBR systems.

Table 5 aims to enlist the advantages and disadvantages of prevalent techniques for micro-contaminant removal from various water/wastewater sources.

## 4. Challenges and Limitations towards the Use of MBR

### 4.1. Membrane Fouling and Its Mitigation in MBRs

Fouling/biofouling is one of the most operational challenges inside membranes. The fouling phenomenon eventuates in clogging the membrane micropores when exposed to wastewater sources with high values of micro-contaminants [148,149,150,151]. One of the major limitations towards the perception of membrane fouling is the complexity in the formation of membrane foulants. Current investigations have proved that the soluble microbial products and extracellular polymeric substances play momentous roles in the rudimentary and final stages of fouling, respectively [152]. Fouling inside the MBRs takes place due to the physicochemical interactions between the biofluid and membrane. When the membrane surface comes into contact with the biological suspension, biosolids precipitation on the membrane surface takes place resulting in a decrement in the amount of flux [153]. Membrane fouling may be divided into two classifications—reversible and irreversible types. Reversible fouling takes place because of weakly bound external substance that precipitate on the surface of the membrane, which results in the formation of a cake layer. In contrast, irreversible fouling can be attributed to the strong attached foulant compounds and pore blocking of the membrane. The controlling process of reversible fouling is done by applying physical cleaning techniques like back flushing, while the cleaning process of irreversible fouling seems to be harder than reversible fouling [154].

### 4.2. Different Techniques to Mitigate Membrane Fouling/Biofouling

In order to mitigate the undesirable fouling phenomenon inside the MBRs, the membrane cleaning process seems to be necessary [155]. Physical, chemical and physicochemical techniques are considered as three prevalent procedures of membrane cleaning. The backwash process is a physical cleaning method, which is just appropriate for hollow fiber membranes where the pumping process of sewage takes place in the reverse direction but it does not have good efficiency for flatsheet membranes. Membrane brushing is identified as another physical cleaning procedure that has the potential of in situ application for a flatsheet membrane. Despite the high speed of utilization, the physical cleaning technique possesses less efficiency than chemical cleaning. Generally, the physical cleaning technique is only able to remove the coarse solid/cake on the membrane’s surface but the chemical cleaning technique possesses a great ability to remove the flocs. The chemical cleaning technique is significantly effective in removing strong particles attached on the membrane’s surface. [156,157,158,159]. If the fouling amount inside the MBRs is not extreme, the in situ cleaning process is generally implemented; if not, the ex situ cleaning process is a suitable choice. Sodium hypochlorite (NaOCl) was the first material industrially applied for membrane cleaning [160,161]. Another momentous parameter, which drastically affects the fouling reduction, is membrane configuration. In an investigation, Katayon et al. corroborated that horizontal membrane configuration caused slower decrement in the flux of permeate flux than the vertical configuration [162]. The well-organized trend of the membrane’s cleaning can considerably enhance the membrane life. Activated carbon (AC) is considered as a promising biofouling reducer in MBRs to extend the membrane life. Biofouling reducers possess great potential to adsorb organic/non-organic micro-contaminants due to having high a surface area that improves the adsorption velocity. Compared to granular AC, powder AC has greater capability to remove low molecular-weight organic micro-contaminants due to having a better surface area [163]. Generally, pore blocking, pore constriction and cake formation are known as the most common mechanisms of membrane fouling occurrence. In many cases, the increment in the powdered activated carbon (PAC)–water contact time applying low operating flux may significantly decline membrane fouling. Moreover, relying on the process configuration and mode of operation, application of a high dose of PAC can reduce membrane fouling. The use of oxidants may possess a brilliant ability to decrease the amount of membrane fouling by modifying the interactions between membrane surface and components of the solution [164]. An appropriate design of bioreactor is of great importance to reduce the occurrence of membrane fouling. Owing to the fact that decreasing the amount of chemical oxygen demand (COD) before exposure to the membrane may reduce membrane fouling, greater sludge retention time (SRT) operation can be effective.

## 5. Conclusions and Future Perspectives

MBRs have emerged as a promising and efficient technology to remove various types of micro-contaminants from aquatic environments. In recent decades, the application of MBRs has illustrated substantial growth. Recent investigations have implied the fact that the MBR-based approaches are of great potential for efficient removal of various types of micro-contaminants such as pesticides, NSAIDs, EDCs, pathogens and cosmetics. One of the most important operational challenges towards the application of MBRs is the occurrence of fouling inside the membrane. Therefore, the development of promising technologies to mitigate its amount inside the MBRs is of great importance. To overcome this challenge, the use of some approaches, such as pretreatment techniques, changing the configuration of the membrane and modification of operating conditions, may be beneficial. Additionally, it is worth pointing out that the cleaning techniques of membranes are essential to mitigate fouling with the aim of ensuring the long-term efficiency of the membrane-based systems for micro-contaminant removal. Despite the lack of efficacy fulfillment of the chemical cleaning approach in the actual operation, more effective cleaning modes should be developed. The main focus must be attributed to the development innovative cleaning approaches to increase the mitigation of fouling in MBRs while consuming less energy, simultaneously. This review paper comprehensively discusses the advantages of MBRs compared to other conventional techniques to remove micro-contaminants in the aquatic environment. Moreover, the removal efficiency of various micro-contaminants using MBRs and other conventional techniques are discussed. Based on the findings, MBRs were significantly efficacious for the removal of various pharmaceutical micro-contaminants from aquatic environment. For example, the average removal percentage of Ibuprofen and Acetaminophen from the aquatic environment using MBRs is more than 90%, which is higher than other removal approaches.

## Figures and Tables

**Table 1:** Detailed information of the most common micro-contaminants in wastewater/surface water sources. Data were provided from the references [6,9,38,39].

Classification	Micro-Contaminant	Average Concentration in Surface Water (ng L^−1^)	Average Concentration in Wastewater (ng L^−1^)
Pesticides	Carbaryl	---	1.6
	Dimethoate	22	---
	Diethyltoluamide (DEET)	135	593
	Diazinon	15	173
Hormone active substances	Estradiol	2	3
	Estrone	2	15
	Nonylphenol	441	267
Pharmaceuticals (NSAID, over the counter (OTC) drugs and veterinary drugs	Diclofenac	65	647
	Erythromycin	25	42
	Ethinylestradiol	5	2
	Ibuprofen	35	394
	Mefenamic acids	7	870
	Metformin	713	10,347
	Naproxen	37	462
	Penicillin V	---	28.7
	Codeine	---	70.6
	Citalopram	---	33.8
	Azithromycin	12	175
	Atenolol	205	843
Detergents and personal care/food products	Gadolinium	---	115
	Buprenorphine	---	3.9
	Maprotiline	---	0.4
	Duloxetine	---	0.1
	Chlorpromazine	---	0.1
	Acesulfame	4010	22,500
	Sucralose	540	4600

**Table 2:** The potential detriments of micro-contaminants on the environment.

Micro-Contaminant	Health Detriments	Ref.
Arsenic	Toxicity for nervous system Muscular cramps Hepatic failure Deficiency of immune system	[51,52]
Mercury	Emotional changes (i.e., irritability) Insomnia Stomach/kidney failure Respiratory toxicity	[53,54]
Nitrate	Methemoglobinemia Brain damage Thyroid disease Neural tube defects	[55,56,57]
Disinfection by-products	Increased risk of bladder cancer Respiratory ailments	[58,59,60]
Fluoride	Skeletal fluorosis Joint stiffness	[61,62,63]
Pesticides	Stinging eyes Rashes Blisters Nausea Dizziness	[64,65,66,67]
Pharmaceutical drugs	Increased risk of various cancers	[68,69,70,71]

**Table 3:** Comprehensive data about the removal process of disparate micro-contaminants from various types of water/wastewater sources applying the G-F technique.

Coagulant/Flocculent	Micro-Contaminant	Source	Removal (%)	Ref.
Ferric chloride/Aluminium sulfate	Ibuprofen	Hospital wastewater	12 ± 4.8	[100]
	Diclofenac		21.6 ± 19.4	
	Naproxen		31.8 ± 10.2	
	Carbamazepine		6.3 ± 15.9	
	Sulfamethoxazole		6 ± 9.5	
	Tonalide		83.4 ± 14.3	
	Galaxolide		79.2 ± 9.9	
Ferric chloride	Bisphenol A	Landfill leachate	20	[101]
	Nonylphenol		90	
Aluminium sulfate	Aldrin	Surface water	46	[102]
	Bentazon		15	
Aluminium sulfate	Estradiol	Drinking water treatment pant	2	[103]
	Estrone		5	
	Progesterone		6	
	Fluoxetine		15	
	Hydrocodone		24	
	Chlordane		25	
	Erythromycin		33	
	DDT		36	
Ferric sulfate	Diclofenac	Lake water with dissolved humic acid	77	[104]
	Ibuprofen		50	
	Bezafibrate		36	
	Carbamazepine		Less than 10	
	Sulfamethoxazole		Less than 10	

**Table 4:** The removal performance of various pharmaceutical micro-contaminants from aquatic environments using the MBR system [123,124,125,126,127,128,129,130].

Classification	Micro–Contaminant	Removal Efficiency (%)
Non–steroidal anti–inflammatory drugs (NSAIDs)	Ibuprofen	73–99.8
	Ketoprofen	3.7–91.9
	Naproxen	40.1–99.3
	Diclofenac	15–87.4
Anti–epileptics/anti–depressant	Acetaminophen	95.1–99.9
	Carbamazepine	42–51
	Diazepam	67
Hormones and EDCs	Estrone	76.9–99.4
	17*β*–estradiol	Higher than 99.4
	17*α*–Ethinylestradiol	0–93.5
	Bisphenol A	88.2–97
Antibiotics	Sulfamethoxazole	20–91.9
	Erythromycin	25.2–90.4
Beta blockers	Atenolol	5–96.9
	Metoprolol	29.5–58.7
Lipid regulator/cholesterol lowering drugs	Bezafibrate	88.2–95.8
	Clofibric acid	25–71
	Gemfibrozil	32.5–85

**Table 5:** Advantages/disadvantages of commonly applied techniques for micro-contaminant removal.

Micro-Contaminants Removal Approach	Positive Points	Drawbacks	Ref.
Coagulation/flocculation	Simplicity of chemicals manufacturing Low cost Decrement in the overall detention time	Toxic sludge disposal The need for skilled operators	[131,132,133,134]
ACA	Simplicity of operation Cost-effectiveness Application in extensive range of pH Good efficiency	Expensive regeneration Lack of regeneration	[135,136,137]
AOP	Fast reaction rate No sludge production	High capital and operating costs Complex chemistry tailored to specific pollutants	[138,139,140]
CW	Low cost Simplicity of operation Efficacious separation of organic components/heavy metals	Limited income potential The need for big surface area of land Risk of ecological exposure	[141,142,143,144]
HRS	High surface area Cheap operation/maintenance Efficacious for handling variable wastewater loading	Hard to scale-up High cost	[145,146,147]
MBRs	Better control of hydrolysates molecular weight Excellent adsorption capacity Great sludge biodegradation	Enzymes’ leakage/deactivation Fouling Concentration polarization	[121,122,140]

## Data Availability

All data are available within the published paper.
